# Exploring the Potential of Peptides Derived from the Coelomic Fluid of *Echinometra lucunter* Targeting Inflammatory Cytokines in Placental Syndromes: In Silico Study

**DOI:** 10.12688/f1000research.166678.2

**Published:** 2025-11-06

**Authors:** Defrin Defrin, Arif Birru, Kevin Cuandra, Ratna Puspita, I Widnyana, Nasya Aqilah, Arzita Fadhilla, Winka Suwardjo, Amanda Amalia, Kalaj Haq, Tirza Amaris, Feroz Aldino, Shabrina Ekitasari, Bernice Sirait, Nathania Kristaningtyas, Rizka Wilanda, Made Ananda

**Affiliations:** 1 Fetomaternal Division, Department of Obstetrics and Gynecology,, Universitas Andalas, Padang, West Sumatra, 25171, Indonesia; 2 Department of Obstetrics and Gynecology, Faculty of Medicine, Universitas Andalas, Padang, West Sumatra, Indonesia; 3 Department of Medicine, Universitas Andalas, Padang, West Sumatera, 25171, Indonesia; 4 Department of Biochemistry, Universitas Pembangunan Nasional Veteran Jakarta, South Jakarta, Jakarta, Indonesia; 5 Department of Medicine, Universitas Pendidikan Ganesha, Singaraja, Bali, Indonesia; 6 Department of Medicine, Universitas Airlangga, Surabaya, East Java, Indonesia; 7 Department of Medicine, Universitas Abdurrab, Pekanbaru, Riau, Indonesia; 8 Department of Pharmacy, Universitas Gadjah Mada, Yogyakarta, Special Region of Yogyakarta, Indonesia; 9 Department of Medicine, Universitas Udayana, Jimbaran, Bali, Indonesia; 10 Department of Physics, Universitas Jenderal Soedirman, Purwokerto, Central Java, Indonesia; 11 Department of Medicine, Universitas Sebelas Maret, Surakarta, Central Java, Indonesia; 12 Department of Medicine, Universitas Padjadjaran, Bandung, West Java, Indonesia; 13 Department of Medicine, Faculty of Medicine Public Health and Nursing, Universitas Gadjah Mada, Yogyakarta, Special Region of Yogyakarta, Indonesia; 14 Department of Medicine, Universitas Katolik Widya Mandala Surabaya, Surabaya, East Java, Indonesia

**Keywords:** Echinometra lucunter¸Inflammatory, In Silico, Peptides, Placental syndromes

## Abstract

**Background:**

Placental syndromes—encompassing pregnancy loss, preterm birth, gestational diabetes mellitus, and preeclampsia—have been strongly linked to dysregulated inflammatory responses at the maternal–fetal interface. This study aims to explore the potential of peptides derived from the coelomic fluid of *Echinometra lucunter* targeting the inflammatory cytokines involved in placental syndromes using in silico approaches.

**Methods:**

The 3D molecular structure of peptides was modeled using the UCSF Chimera application. The absorption, distribution, metabolism, and excretion (ADMET) properties were analysed using SwissADME and the ProTox web server. The PerMM web server was used to estimate membrane permeability. Crystal structures of target proteins—including c-Met, Interleukin-1β (IL-1β), Interleukin-10 (IL-10), Macrophage Migration Inhibitory Factor (MIF), Platelet-Derived Growth Factor (PDGF), and TNF-Related Apoptosis-Inducing Ligand (TRAIL)—were obtained from the Protein Data Bank Japan (PDBj). Molecular docking and structural visualization were conducted using Molecular Operating Environment (MOE) software, while molecular dynamics simulations were subsequently performed using the YASARA Dynamics software to assess the stability and conformational behavior of the ligand-receptor complexes.

**Results:**

Peptide A was selected based on favorable ADMET properties. Molecular docking results revealed that Peptide A exhibits low binding affinities toward pro-inflammatory mediators, including TRAIL (–10.02 kcal/mol), MIF (–9.32 kcal/mol), IL-1β (–8.29 kcal/mol), and PDGF (–10.44 kcal/mol). Furthermore, Peptide A showed potential agonistic interactions with IL-10 (–10.26 kcal/mol) and c-Met (–9.27 kcal/mol), indicating a possible role in restoring anti-inflammatory and angiogenic signaling. Molecular dynamics simulations supported the stability of the peptide–receptor complexes.

**Conclusions:**

Peptide A holds promise as a dual-function therapeutic agent in placental syndromes. However, experimental validation is necessary to confirm its biological efficacy and safety.

## Introduction

Placental syndromes represent a cluster of pregnancy complications that arise due to functional and structural defects of the placenta. These include a range of pathological processes such as inflammation, oxidative stress, endothelial dysfunction, impaired trophoblast invasion, abnormal angiogenesis, vascular fibrosis, and trophoblast apoptosis. ^1– 4^ As the placenta is the only transient organ in humans and plays a vital role in maternal-fetal nutrient and gas exchange, its dysfunction can lead to serious pregnancy complications, including preeclampsia, chronic hypertension during pregnancy, gestational diabetes mellitus (GDM), miscarriage, and preterm labor. ^4, 5^ Given the severity of these conditions, especially in contributing to maternal and neonatal morbidity and mortality, a deeper molecular understanding of placental syndromes is urgently required.

Recent studies have focused on key molecular mediators involved in these pathologies. In preeclampsia, Interleukin-10 (IL-10) acts as a potent anti-inflammatory cytokine crucial for immune tolerance during pregnancy. ^6^ In contrast, Hepatocyte Growth Factor (HGF) promotes trophoblast invasion via the c-Met receptor, contributing to adequate placental development. ^7^ In chronic hypertension associated with pregnancy, Platelet-Derived Growth Factor (PDGF) and its receptors (PDGF-R) are central to vascular remodeling and are frequently dysregulated. ^8^ In GDM, Macrophage Migration Inhibitory Factor (MIF) has been implicated in placental inflammation, ^3^ while in pregnancy loss, Interleukin-1β receptor (IL-1βR) mediates pro-inflammatory pathways that impair trophoblastic function. ^6^ Furthermore, Tumour necrosis factor-related apoptosis-inducing ligand (TRAIL) signalling via DR4 and DR5 receptors promotes trophoblast apoptosis, contributing to preterm birth. ^9^

Despite growing insights, therapeutic approaches targeting these pathways remain limited. Conventional therapies, such as curcumin (an IL-10 activator), crizotinib (a c-Met agonist), imatinib (a PDGF-R inhibitor), and ISO-1 (an MIF inhibitor), show promise; however, their specificity, safety, and efficacy during pregnancy remain suboptimal. ^10– 12^ Exploring natural compounds, especially peptide-based drugs derived from marine organisms, *Echinometra lucunter*, presents a compelling alternative. These natural peptides are highly specific and less toxic, making them suitable candidates for treating placental syndromes. ^13^

However, current understanding of their mechanisms of action and protein-target interactions remains limited. Advances in bioinformatics and in silico methodologies have emerged as powerful tools to investigate ligand-receptor interactions at the atomic level. ^14, 15^ Therefore, this study aim to explore the interaction of peptides derived from *E. lucunter* targeting the inflammatory cytokines involved in placental syndromes. By comparing natural peptide ligands with existing synthetic control drugs, this research seeks to identify novel peptide candidates with favourable binding affinity and stability profiles. This study is also expected to deepen our understanding of placental syndromes and provide a foundation for the development of peptide-based therapeutics to improve maternal and fetal health outcomes.

## Methods

### Ligand preparation

A total of 12 peptide sequences identified from the coelomic fluid of *Echinometra lucunter* were retrieved from a previously conducted peptidomics profiling study. ^16^ The three-dimensional structures of the peptides were modelled using UCSF Chimera version 1.17.1 ( Table 1). Native co-crystallised ligands from each protein target were extracted using BIOVIA Discovery Studio 2024 to serve as control ligands. All ligands underwent refinements using the Molecular Operating Environment (MOE) application (version 2022.02), ensuring an optimised geometry with a Root Mean Square (RMS) gradient convergence threshold set to 0.001 kcal/mol/Å ^2^. ^17^

**Table 1.** Peptide sequences.

Peptide Code	Peptide Sequence
Peptide A	FLMLVDGH
Peptide B	LASVP
Peptide C	LGQLT
Peptide D	LGSR
Peptide E	LLHA
Peptide F	LPPP
Peptide G	PPVF
Peptide H	REGSPDLR
Peptide I	TGGGLPV
Peptide J	VEGSLVLR
Peptide K	VTTKH
Peptide L	VFMA

### Protein preparation

The three-dimensional crystallographic structures of six key protein targets—c-Met, Interleukin-1β (IL-1β), Interleukin-10 (IL-10), Macrophage Migration Inhibitory Factor (MIF), Platelet-Derived Growth Factor (PDGF), and TNF-Related Apoptosis-Inducing Ligand (TRAIL)—were retrieved from the Protein Data Bank ( https://www.rcsb.org) ( Table 2). These proteins were selected based on their well-documented involvement in the pathogenesis of placental syndromes, further substantiated by Mendelian randomization findings from a previous study. ^18^ All target proteins were prepared, neutralized, and refined to a Root Mean Square (RMS) gradient of 0.001 kcal/mol/Å ^2^ using the MOE software (version 2022.02). ^17^

**Table 2.** The PDB IDs of target proteins.

Target protein	PDB ID
c-Met	2WGJ
IL-1β	5R8Q
IL-10	1INR
MIF	1LJT
PDGF	3MJK
TRAIL	1D4V

### Absorption, distribution, metabolism, excretion, and toxicity (ADMET) prediction

Absorption, distribution, metabolism, excretion, and toxicity (ADMET) were conducted for all E. lucunter peptides. Canonical SMILES notations of each peptide were submitted to the SwissADME webserver ( http://www.swissadme.ch/index.php; accessed April 21, 2025) to predict ADME properties. This evaluation aimed to predict the potential impacts of the peptides on human health by assessing key pharmacokinetic parameters. Specifically, the analysis included predictions of human intestinal absorption, Caco-2 permeability, blood-brain barrier (BBB) permeability, cytochrome P450 enzyme 2D6 interactions (CYP2D6 substrate and inhibitor), and water solubility (Log S (ESOL)). ^19^

Toxicity predictions were carried out using the ProTox-III webserver ( https://tox.charite.de/protox3/; accessed April 21, 2025). The ProTox classified the peptides into six toxicity categories based on predicted LD _50_ values (mg/kg BW): Class I (LD _50_ ≤ 5 mg/kg BW; fatal if swallowed), Class II (5 < LD _50_ ≤ 50 mg/kg BW; fatal if swallowed), Class III (50 < LD _50_ ≤ 300 mg/kg BW; toxic if swallowed), Class IV (300 < LD _50_ ≤ 2000 mg/kg BW; harmful if swallowed), Class V (2000 < LD _50_ ≤ 5000 mg/kg BW; may be harmful if swallowed), and Class VI (LD _50_ > 5000 mg/kg BW; non-toxic). ^20^

### Molecular docking

Molecular docking was performed using the MOE application (version 2022.02). The binding site was determined based on the interactions of the native ligand retrieved from the crystallographic structure deposited in the PDB database and further refined using the Site Finder tool in MOE to ensure accurate pocket identification. To validate the docking protocol, a redocking procedure was conducted by re-docking the native ligand into the defined binding site. The resulting Root Mean Square Deviation (RMSD) value of less than 2 Å confirmed the reliability and reproducibility of the docking method. Ligand-protein interactions were analysed and evaluated based on binding affinity (kcal/mol) and Root Mean Square Deviation (RMSD) values. The peptide was selected based on the RMSD values below 2.5 Å and lower binding affinity (more negative values) compared to control ligands. Structural visualisation in both 2D and 3D formats further validated the spatial proximity and interaction patterns between peptides and their protein targets, supporting their candidacy as selective peptides. ^17^

### Molecular dynamics simulation

Molecular dynamics (MD) simulations were conducted using YASARA Dynamics version 4.3.13. Selected protein–peptide complexes were imported into the application by selecting the “Set Target” and “Macro & Movie” options from the Options menu. The simulation environment was configured under physiological conditions, with a temperature of 310 K, atmospheric pressure of 1 atm, pH 7.4, and a 0.9% NaCl concentration. The AMBER14 force field was applied, and the system was enclosed within a cubic periodic boundary cell extended by 10 Å. The total simulation time was set to 50,000 picoseconds (50 ns). Post-simulation, the Root Mean Square Deviation (RMSD) of the Cα atoms was calculated using the MD_analysis macro to evaluate structural stability over time. The results of the MD simulations were visualised using ORIGINPro 2024 (OriginLab, Massachusetts, United States). ^21^

### Peptide membrane permeability prediction

The membrane permeability of the selected peptides was predicted using the PerMM (Permeability of Molecules across Membranes) web server. ^22^ Simulations were performed under physiological conditions (pH 7.4, 298 K temperature) using the “drag” optimization algorithm. The output included peptide transfer energy profiles as a function of their position relative to the membrane centre, along with membrane binding affinity (ΔG, kcal/mol) and membrane permeability coefficients (logPerm). ^22^

## Results

### Absorption, distribution, metabolism, excretion, toxicity (ADMET) prediction

The in silico ADME analysis revealed that most of the evaluated peptides (Peptides A–L) exhibited favourable pharmacokinetic profiles. In terms of absorption, seven peptides (Peptides A, E, F, I, J, K, and L) showed high predicted human intestinal absorption (HIA > 0.80) ( Table 3). The remaining peptides demonstrated moderate absorption values, which are still acceptable for further development. Despite favourable absorption, all peptides were predicted to have low Caco-2 permeability, which may present a limitation in intestinal permeability; however, it does not exclude their bioavailability potential, particularly when alternative delivery systems are considered.

**Table 3.** The absorption, distribution, metabolism, and excretion results of peptides derived from the coelomic fluid of *E. lucunter.*

Peptide	Human intestinal absorption	Caco-2 permeability	BBB Permeant	CYP2D6 substrate	CYP2D6 inhibitor	OCT2 inhibitor	Log s (ESOL)
Peptide A	0.8089 (High)	0.7874 (Low)	No	No	No	No	Soluble
Peptide B	0.7936 (Moderate)	0.7083 (Low)	No	No	No	No	Very soluble
Peptide C	0.6539 (Moderate)	0.7799 (Low)	No	No	No	No	Highly soluble
Peptide D	0.7518 (Moderate)	0.8169 (Low)	No	No	No	No	Very soluble
Peptide E	0.8676 (High)	0.8126 (Low)	No	No	No	No	Very soluble
Peptide F	0.8761 (High)	0.7537 (Low)	Yes	No	No	No	Very soluble
Peptide G	0,6394 (Moderate)	0,7806 (Low)	No	No	No	No	Very soluble
Peptide H	0,5207 (Moderate)	0,7247 (Low)	No	No	No	No	Highly soluble
Peptide I	0,9386 (High)	0,7648 (Low)	No	No	No	No	Soluble
Peptide J	0,9340 (High)	0,6751 (Low)	No	No	No	No	Soluble
Peptide K	0,9210 (High)	0,7877 (Low)	No	No	No	No	Soluble
Peptide L	0,9589 (High)	0,6558 (Low)	No	No	No	No	Very soluble

In terms of distribution, only Peptide F was predicted to cross the blood–brain barrier (BBB), indicating potential utility in targeting the central nervous system (CNS). The other peptides were non-permeant to the BBB, which could be advantageous for avoiding undesired CNS-related side effects in peripheral applications. None of the peptides were identified as substrates or inhibitors of the cytochrome P450 2D6 (CYP2D6) enzyme, indicating a low risk of CYP-mediated metabolism or drug–drug interactions—an important consideration for safety and consistency in pharmacokinetics. Similarly, none were predicted to inhibit the organic cation transporter 2 (OCT2), suggesting that these peptides are unlikely to interfere with renal excretion pathways. Regarding solubility, all peptides displayed good aqueous solubility as predicted by the ESOL model. Peptides C and H were classified as highly soluble, Peptides B, D, E, F, G, and L as very soluble, and Peptides A, I, J, and K as soluble. This favourable solubility profile supports their potential for oral and parenteral formulation development.

The toxicological assessment of peptides derived from *E. lucunter* exhibited a range of toxicity classifications from Class III (toxic) to Class VI (non-toxic) ( Table 4). Among them, peptide L was categorised as non-toxic (Class VI) with an LD _50_ of 6,000 mg/kg BW, whereas peptide G was classified as toxic (Class III) with an LD _50_ of 210 mg/kg BW. Five compounds—peptide B (2,000 mg/kg BW), peptide C (1,190 mg/kg BW), peptide D (836 mg/kg BW), peptide H (2,000 mg/kg BW), and peptide J (2,000 mg/kg BW)—were categorised to Class IV (harmful). Another five compounds—peptide A (2,400 mg/kg BW), peptide E (5,000 mg/kg BW), peptide F (2,500 mg/kg BW), peptide I (5,000 mg/kg BW), and peptide K—were categorised under Class V (may be harmful). Similarity scores ranged from 65.77% to 100%, with prediction accuracy above 68% for all compounds.

**Table 4.** The predicted LD _50_ and toxicity class of peptides derived from the coelomic fluid of *E. lucunter.*

Compound	Predicted LD _50_	Predicted toxicity class	Average similarity	Prediction accuracy
Peptide A	2,400 mg/kg	Five (possibly hazardous)	71,26%	69,26%
Peptide B	2,000 mg/kg	Four (harmful)	80,43%	70,97%
Peptide C	1,190 mg/kg	Four (harmful)	100%	100%
Peptide D	836 mg/kg	Four (harmful)	75,27%	69,26%
Peptide E	5,000 mg/kg	Five (possibly hazardous)	95,05%	72,90%
Peptide F	2,500 mg/kg	Five (possibly hazardous)	79,62%	69,26%
Peptide G	210 mg/kg	Three (toxic)	88,78%	70,97%
Peptide H	2,000 mg/kg	Four (harmful)	65,77%	68,07%
Peptide I	5,000 mg/kg	Five (possibly hazardous)	73,85%	69,26%
Peptide J	2,000 mg/kg	Four (harmful)	71,62%	69,26%
Peptide K	6,000 mg/kg	Six (nontoxic)	68,04%	68,07%
Peptide L	3,000 mg/kg	Five (possibly hazardous)	83,08%	70,97%

Organ toxicity assessment revealed that the majority of peptides were classified as inactive across five organ toxicity parameters: hepatotoxicity, carcinogenicity, immunotoxicity, mutagenicity, and cytotoxicity. Specifically, all peptides, except peptide C, were predicted to be inactive across all parameters ( Table 5). In contrast, peptide C was predicted to be active in terms of hepatotoxicity and immunotoxicity.

**Table 5.** The organ target toxicity prediction results for peptides derived from the coelomic fluid of *E. lucunter.*

Compounds	Organ toxicity				
	Hepato-toxicity	Carcino-toxicity	Immuno-toxicity	Muta-genicity	Cyto-toxicity
Peptide A	inactive	inactive	inactive	inactive	inactive
Peptide B	inactive	inactive	inactive	inactive	inactive
Peptide C	active	inactive	active	inactive	inactive
Peptide D	inactive	inactive	inactive	inactive	inactive
Peptide E	inactive	inactive	inactive	inactive	inactive
Peptide F	inactive	inactive	inactive	inactive	inactive
Peptide G	inactive	inactive	inactive	inactive	inactive
Peptide H	inactive	inactive	inactive	inactive	inactive
Peptide I	inactive	inactive	inactive	inactive	inactive
Peptide J	inactive	inactive	inactive	inactive	inactive
Peptide K	inactive	inactive	inactive	inactive	inactive
Peptide L	inactive	inactive	inactive	inactive	inactive

### Molecular docking

***TRAIL***

**Figure 1.**
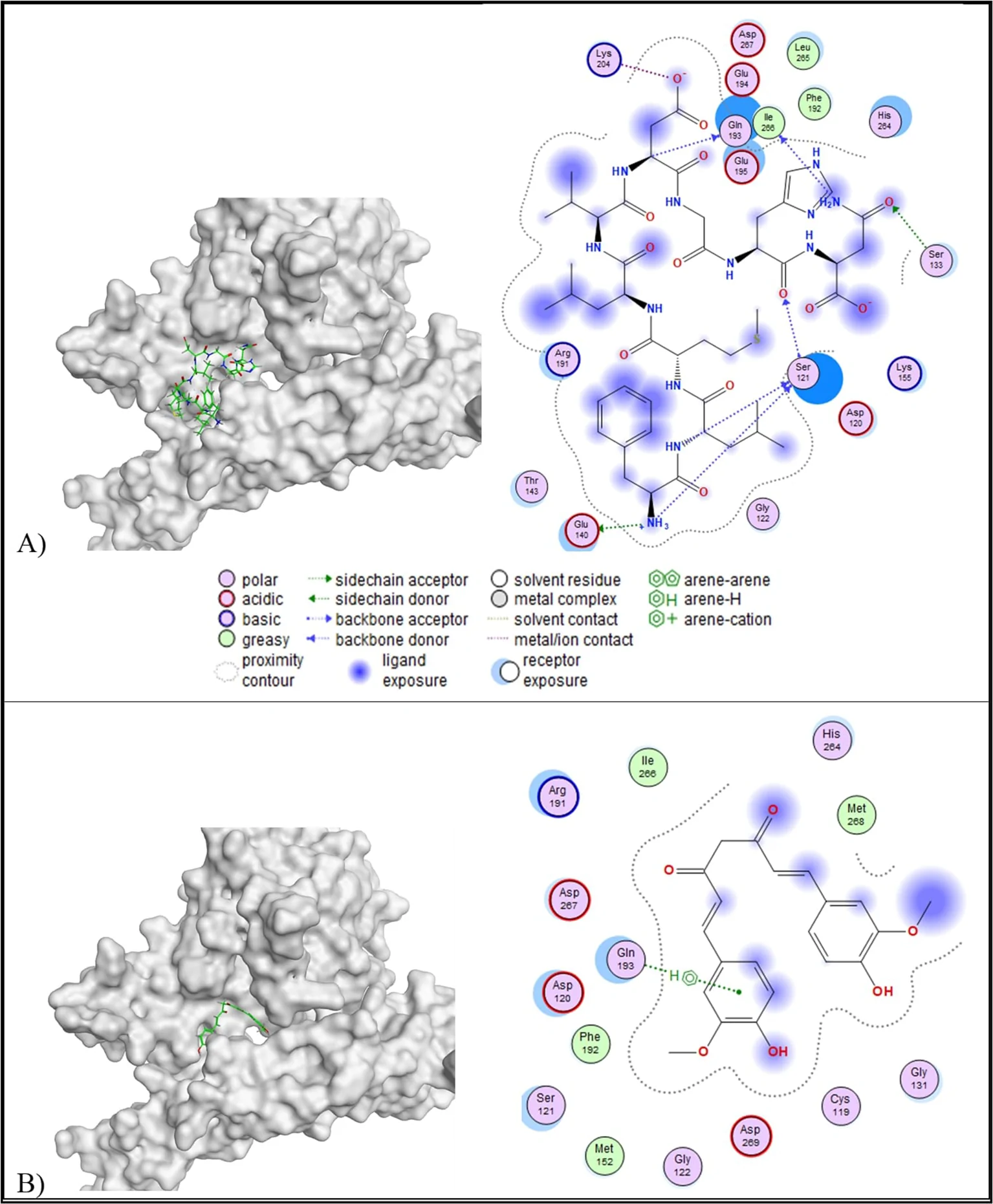
Two- and three-dimensional visualizations of the TRAIL complex with A) Peptide A and B) control ligand.

Based on the molecular docking result, all peptides, except peptide H, showed lower binding affinity than the control ligand (-6.96 kcal/mol) and RMSD values below 2.5 Å. Although peptide H (REGSPDLR) exhibited the lowest binding affinity (-10.72 kcal/mol) among the other, peptide H has RMSD values more than 2.5 Å (2.68 Å) ( Table 5). Therefore, peptide A (FLMLVDGH) was selected based on its favourable binding affinity (-10.02 kcal/mol) and stable conformation (RMSD = 2.39 Å). In addition to docking parameters, peptide A also exhibited a low toxicity profile (toxicity class V, LD50 = 2,400 mg/kg BW) and a favourable ADME profile. The 2D visualisation of molecular docking results revealed that peptide A formed more interactions with amino acid residues of TRAIL than the control ligand. Peptide A formed six interactions with the amino acid residues of TRAIL, consisting of one acidic hydrophilic (Glu140), one basic hydrophilic (Lys204), three polar hydrophilic (Ser121, Ser133, Gln193), and one greasy hydrophobic (Ile266) interaction ( Figure 1). In comparison, the control ligand formed one polar hydrophilic interaction (Gln193) with the amino acid residues of TRAIL. A similar interaction at the active site of TRAIL (Gln193) was observed between peptide A and the control ligand.

**Figure 2.**
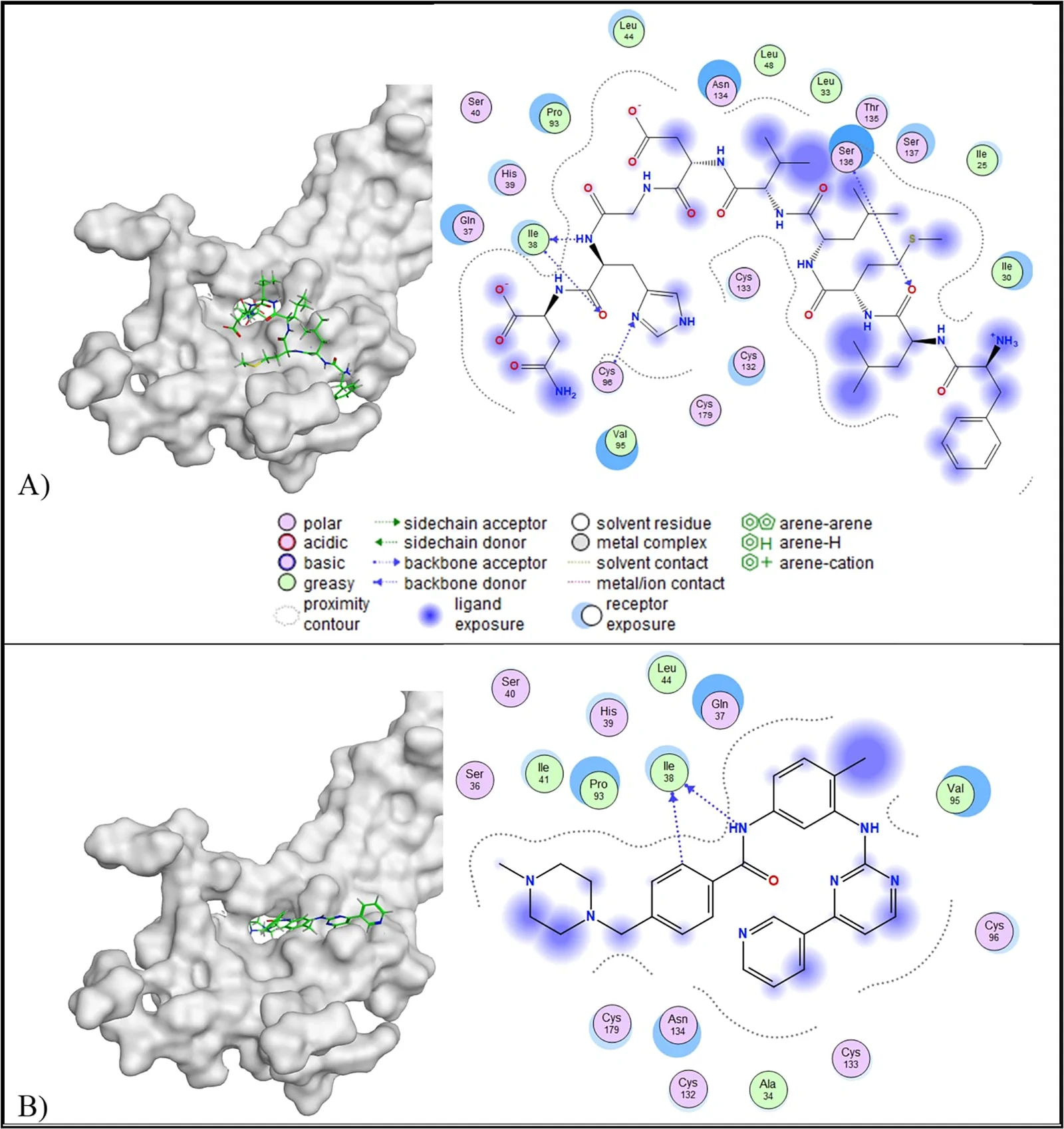
Two- and three-dimensional visualizations of the PDGF complex with A) Peptide A and B) control ligand.

Molecular docking results revealed that peptides A, B, C, G, H, I, and J have lower binding affinity than the control ligand and an RMSD value lower than 2.5 Å ( Table 5). Among the favourable peptides, peptide A has the lowest binding affinity (-10.44 kcal/mol). Based on 2D interaction analysis, peptide A formed four interactions with the PDGF, consisting of two polar interactions with Asn134 and Ser136, and a greasy hydrophobic interaction with Ile38 ( Figure 2). Meanwhile, the control ligand formed one greasy hydrophobic interaction at Ile38. Both peptide A and the control ligand shared a similar interaction at Ile38 at the active site of PDGF.

***MIF***

**Figure 3.**
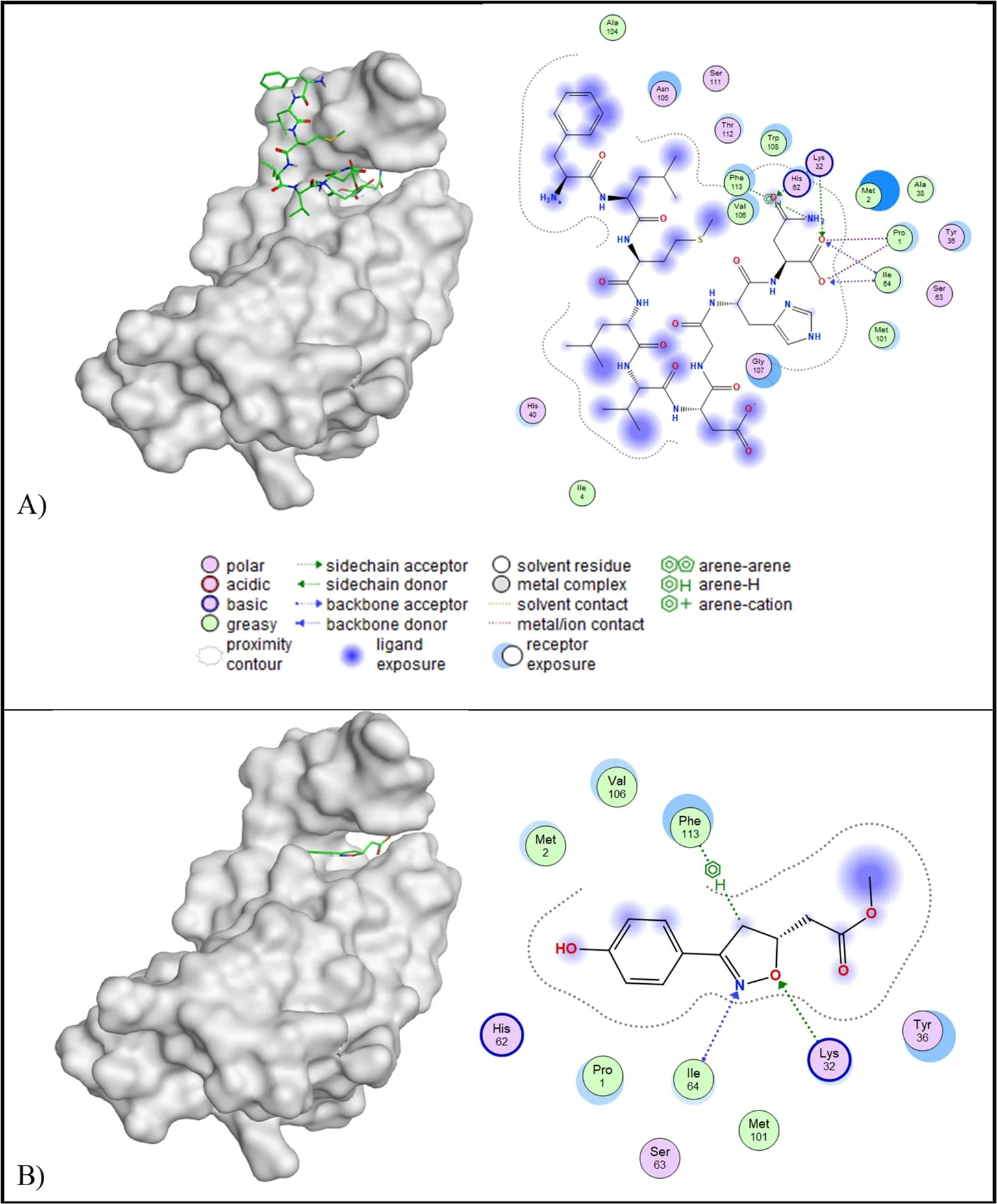
Two- and three-dimensional visualizations of the MIF complex with A) Peptide A and B) control ligand.

All peptides of E. lucunter showed lower binding affinity than the control ligand (5.95 kcal/mol). In terms of RMSD values, all peptides, except peptide H, met the criteria of an RMSD value below 2.5 Å ( Table 5). Peptide A was selected due to its lowest binding affinity (-9.32 kcal/mol) compared to the other favourable peptides. Based on 2D interaction analysis, peptide A formed three interactions with the MIF active site: one basic hydrophilic interaction (Lys32) and three greasy hydrophobic interactions (Phe113, Ile64, and Pro1). On the other side, the control ligand created one basic interaction (Lys32) and two greasy hydrophobic interactions (Ile64 and Phe113). Notably, both peptide A and the control ligand shared common interaction sites at Lys32, Ile64, and Phe113, indicating a similar binding orientation within the MIF active site ( Figure 3).

***IL-10***

**Figure 4.**
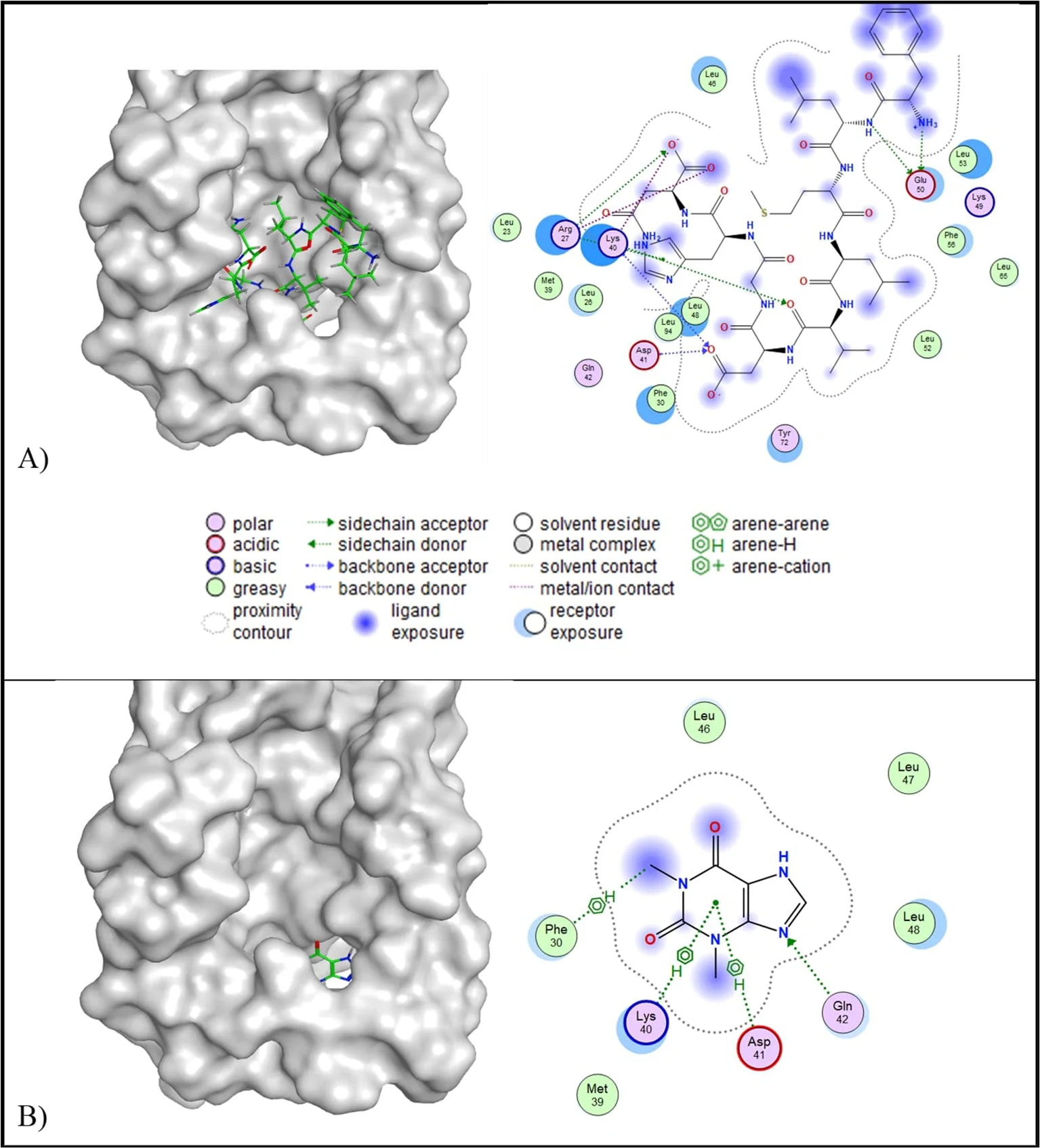
Two- and three-dimensional visualizations of the IL-10 complex with A) Peptide A and B) control ligand.

In the context of IL-10 protein targeting, all candidate peptides demonstrated RMSD values below 2.5 Å and lower binding affinities compared to the control ligand (−4.44 kcal/mol). Among them, peptide J exhibited the lowest binding affinity (−10.54 kcal/mol); however, peptide J also presented a relatively high predicted toxicity (toxicity class IV; LD _50_ = 2,000 mg/kg BW). Due to safety considerations, peptide A was selected instead, as it exhibited a lower predicted toxicity (toxicity class V; LD _50_ = 2,400 mg/kg BW), while maintaining a lower binding affinity (−10.26 kcal/mol) and an RMSD value of 2.41 Å ( Table 5). Two-dimensional interaction analysis revealed that peptide A formed four hydrophilic interactions with IL-10, involving two acidic residues (Asp41 and Glu50) and two basic residues (Arg27 and Lys40). In contrast, the control ligand interacted with four residues, including Asp41 (acidic), Lys40 (basic), Gln42 (polar), and Phe30 (greasy). Notably, both peptide A and the control ligand shared interaction sites at Asp41 and Lys40, suggesting potential overlap in binding specificity ( Figure 4).

***IL-1β***

**Figure 5.**
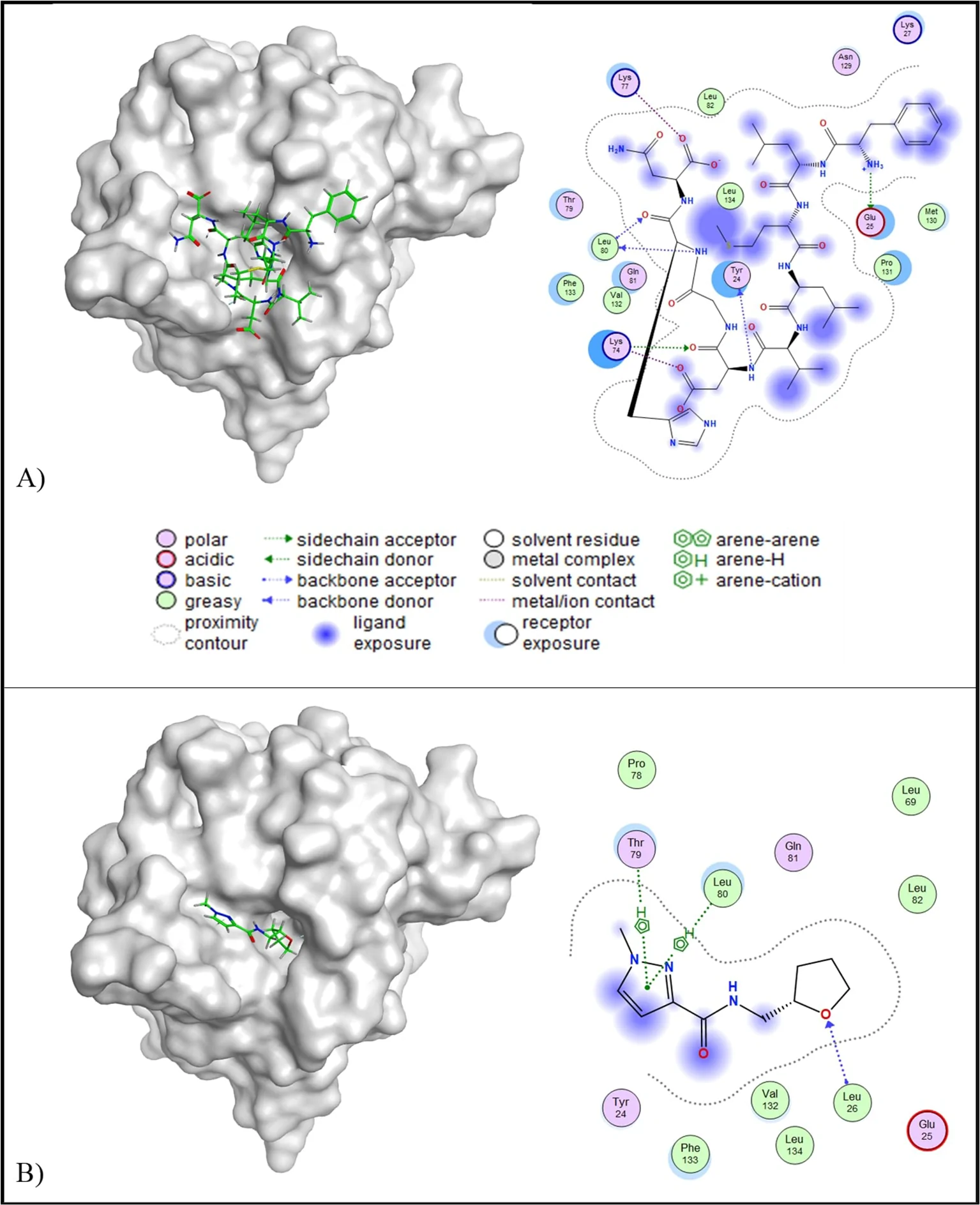
Two- and three-dimensional visualizations of the IL-1β complex with A) Peptide A and B) control ligand.

Molecular docking results indicated that all of the peptides have lower binding affinity than the control ligand (-6.23 kcal/mol) and RMSD below 2.5 Å ( Table 5). Peptide A was selected for further analysis due to its potential as a multi-target inhibitor, targeting TRAIL, PDGF, MIF, and IL-10. Peptide A has a binding affinity value of -8.29 kcal/mol and RMSD value of 1.94 A. Peptide A engages IL-1β through five key interactions: one acidic hydrophilic interaction (Glu25), two basic hydrophilic interactions (Lys74 and Lys77), one polar interaction (Tyr24), and one hydrophobic interaction (Leu80). In contrast, the control ligand formed fewer interactions, including a polar hydrophilic interaction (Thr79) and two hydrophobic interactions (Leu26 and Leu80) ( Figure 5). Importantly, both peptide A and the control ligand shared a similar hydrophobic interaction with Leu80.

***c-Met***

**Figure 6.**
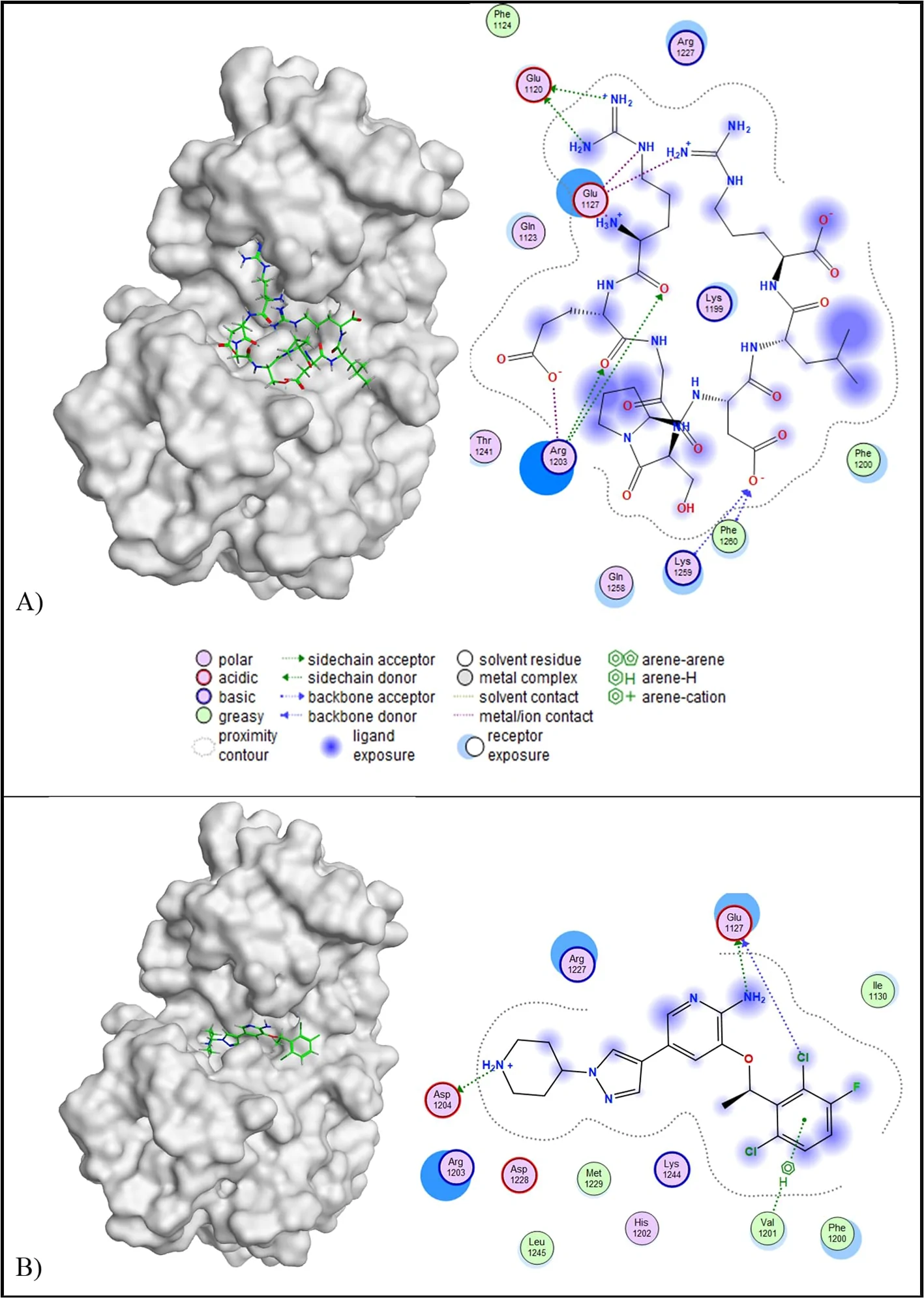
Two- and three-dimensional visualizations of the c-Met complex with A) Peptide A and B) control ligand.

Molecular docking analysis revealed that ten out of twelve peptides exhibited root mean square deviation (RMSD) values lower than 2.5 Å and lower binding affinities compared to the control ligand (−6.87 kcal/mol). Among the favourable peptides, peptide A demonstrated the most promising interaction, with a binding affinity of −9.27 kcal/mol and an RMSD of 1.78 Å ( Table 5). Peptide A was therefore selected for further analysis due to its potential as a multi-target inhibitor across five additional protein targets. Two-dimensional interaction analysis showed that peptide A established five molecular interactions with the target protein: Glu1120, Glu1127, Arg1203, Lys1259, and Phe1260. These interactions included two acidic hydrophilic (Glu1120 and Glu1127), two basic hydrophilic (Arg1203 and Lys1259), and one hydrophobic (Phe1260) contact. In contrast, the native c-Met inhibitor formed only three interactions: Glu1127, Asp1204, and Val1201, comprising two acidic hydrophilic and one hydrophobic interaction. Notably, both peptide A and the c-Met inhibitor shared a similar interaction at residue Glu1127. The three-dimensional conformation of the c-Met-peptide A complex is illustrated in Figure 6.

### Permeability of Molecules through Membranes (PerMM) Analysis

**Figure 7.**
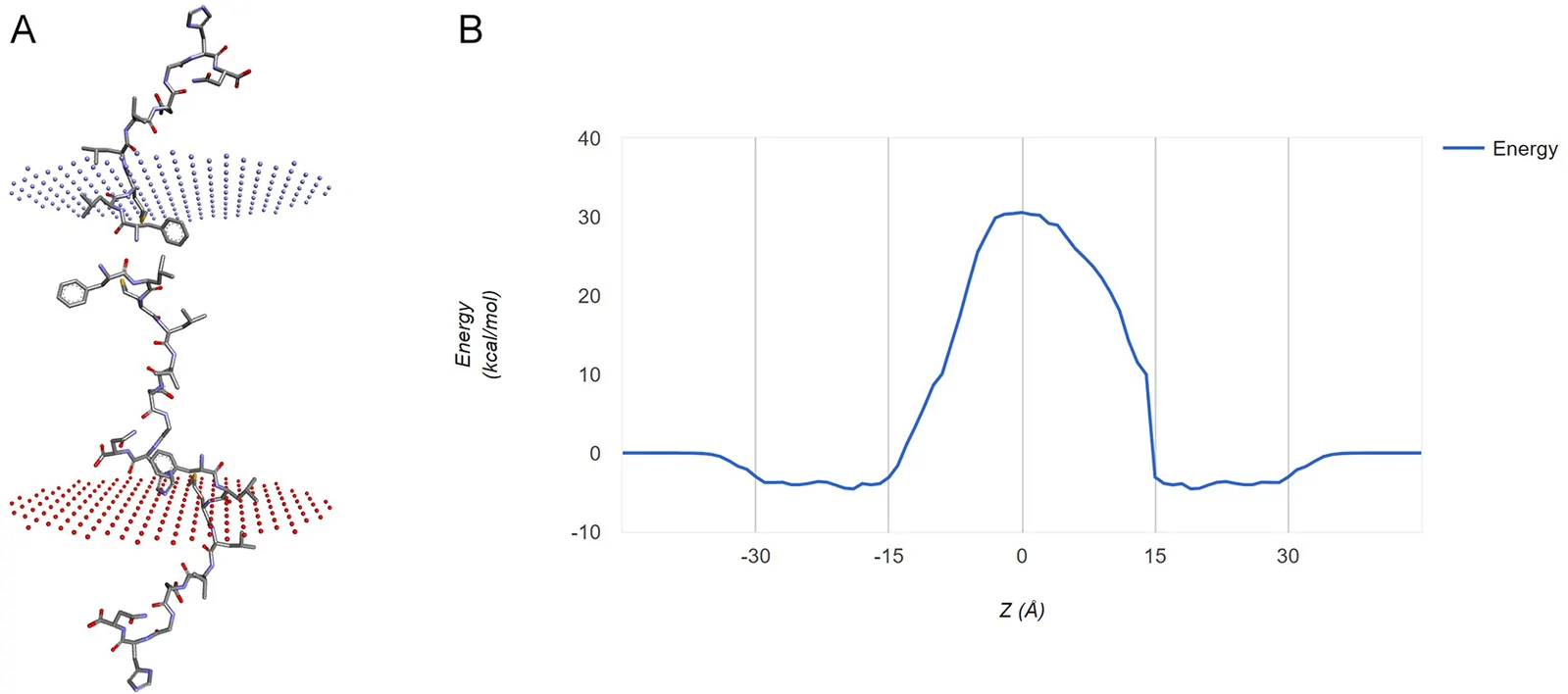
The membrane permeability results of Peptide A. (A) Interaction of Peptide A with the DOPC bilayer. (B) Energy profile of Peptide A along the Z-axis (kcal/mol).

PerMM (Permeability of Molecules through Membranes) analysis of Peptide A (FLMLVDGHN) showed that this peptide exhibited the strongest membrane-binding affinity among the tested candidates, with a calculated free energy of interaction (ΔG) of -4.63 kcal/mol with a DOPC lipid bilayer. Despite this favourable membrane anchoring, Peptide A demonstrated extremely low permeability, indicated by a log P_BLM value of -21.36. Additionally, the energy barrier at the bilayer centre reached a peak of +30.57 kcal/mol, further confirming its inability to translocate across the membrane passively ( Figure 7).

### Molecular dynamics simulations

**Figure 8.**
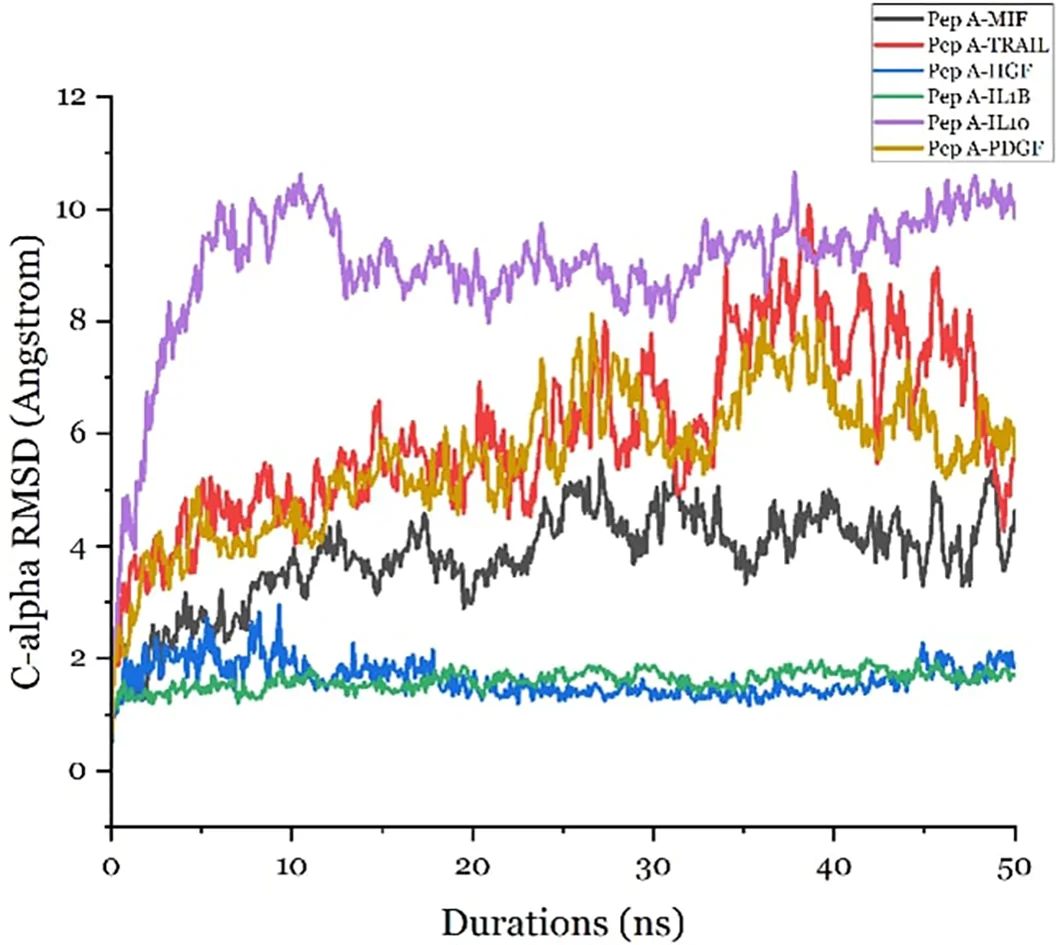
Molecular dynamics simulation results of Peptide A with MIF, TRAIL, c-Met/HGF, IL-1β, IL-10, and PDGF.

Molecular dynamics (MD) simulations of six receptor–ligand complexes revealed that Peptide A forms stable interactions with both IL-10 and IL-1β. This stability was evidenced by Cα backbone root mean square deviation (RMSD) values that consistently remained below 2 Å throughout the 50-nanosecond simulation trajectories. The simulation timeframe falls within the widely accepted 50 ns window for evaluating ligand–receptor complex stabilization, ^23^ and the resulting RMSD values fall well within the established threshold of <3 Å, indicating productive and stable interactions. ^24^ Among the targets assessed, IL-10 and IL-1β displayed the highest binding stability, suggesting their strong potential for functional modulation by Peptide A ( Figure 8).

## Discussion

Peptide A (FLMLVDGHN) was chosen as a lead candidate for placental syndrome therapeutic agents based on its balanced pharmacokinetic and safety profile, despite some limitations in membrane permeability. It showed high predicted human intestinal absorption (HIA = 0.8089), indicating good potential for oral uptake, even though its Caco-2 permeability was low. This suggests that with an appropriate drug delivery system, such as encapsulation or carrier-based transport, its bioavailability could still be optimized. ^19^ The peptide also demonstrated good water solubility, making it suitable for formulation development. Importantly, it was not predicted to interact with major metabolic enzymes (CYP2D6) or renal transporters (OCT2), which lowers the risk of metabolic instability or drug–drug interactions. Although PerMM analysis showed that Peptide A binds strongly to membrane surfaces (ΔG = -6.76 kcal/mol), it also indicated very poor passive translocation due to a high energy barrier (+24.96 kcal/mol), suggesting that while the peptide may associate with membranes, it is unlikely to cross them without assistance. ^22^ From a safety perspective, Peptide A was classified in toxicity Class V, meaning it may be harmful only at relatively high doses (LD _50_ = 2,400 mg/kg BW), and it showed no predicted organ-specific toxicities. ^20^

Chronic inflammation at the maternal–fetal interface is a hallmark of various placental syndromes, including preeclampsia, gestational diabetes mellitus (GDM), and recurrent miscarriage. Pro-inflammatory mediators such as TRAIL, MIF, IL-1β, and PDGF play key roles in amplifying immune activation, disrupting vascular remodeling, and impairing trophoblast function. ^18^ TRAIL, while physiologically involved in immune tolerance, can be pathologically overexpressed in miscarriage, contributing to immune-mediated tissue injury. ^25– 28^ Similarly, macrophage migration-inhibitory factor (MIF), an evolutionarily conserved cytokine, is significantly upregulated in GDM. Elevated placental and circulating MIF levels correlate with insulin resistance and placental inflammation. ^29, 30^ Another critical target is interleukin-1β (IL-1β), a cytokine strongly implicated in embryo implantation failure and miscarriage. ^31^ Excessive IL-1β expression in the decidua has been documented in patients with recurrent pregnancy loss. ^31, 32^ In preeclampsia, increased PDGF levels are associated with vascular dysfunction and inadequate spiral artery remodeling. ^33^ Our study revealed that Peptide A, derived from the coelomic fluid of *E. lucunter*, exhibits a specific multitarget inhibitor potential against several key mediators (TRAIL = -10.02 kcal/mol, MIF = -9.32 kcal/mol, IL-1β = -8.29 kcal/mol, and PDGF = -10.44 kcal/mol) of inflammation and vascular dysfunction in placental syndromes, as proven by a lower binding affinity value than the control ligands.

While excessive inflammation contributes to placental dysfunction, impaired anti-inflammatory signaling further exacerbates disease severity. Interleukin-10 (IL-10), a pleiotropic anti-inflammatory cytokine, plays a central role in maintaining immune tolerance during pregnancy by limiting Th1 responses, antigen presentation, and endothelial activation. ^18^ In preeclampsia, IL-10 expression is significantly reduced in maternal serum and placental tissues, contributing to increased TNF-α, endothelial dysfunction, and placental hypoperfusion. ^34, 35^ IL-10-deficient animal models recapitulate these clinical features, underscoring its protective role in gestation. ^36^ In our study, Peptide A was found to bind with low binding affinity (–10.26 kcal/mol) to the IL-10 receptor (IL-10R), suggesting potential agonist activity. The docking interaction mimics native IL-10 binding, supporting the hypothesis that Peptide A may promote downstream anti-inflammatory signaling. This aligns with previous in vivo model studies, which indicate that IL-10 supplementation restores vascular function, reduces hypertension, and improves fetal growth in preeclampsia. ^37^ A similar mechanism may apply to hepatocyte growth factor (HGF), a trophic factor essential for placental angiogenesis via the c-Met receptor. In preeclampsia, downregulated HGF/c-Met signaling impairs trophoblast invasion and spiral artery remodeling. ^38^ Peptide A bound to c-Met with moderate affinity (–9.27 kcal/mol), raising the possibility of agonist-like enhancement of HGF activity. Thus, by mimicking key anti-inflammatory and angiogenic signals, Peptide A emerges as a dual-function agent that may help restore immune and vascular homeostasis in hypertensive pregnancy disorders.

Root mean square deviation (RMSD) analysis of Cα backbone atoms was performed through 50 ns molecular dynamics simulations to evaluate the conformational stability and receptor engagement of Peptide A in complex with six target proteins. The Peptide A-IL-10 and Peptide A-IL-1β complexes exhibited RMSD values consistently below 2 Å, indicating minimal structural fluctuation and high stability throughout the simulation trajectory. RMSD thresholds below 3 Å are widely regarded as indicative of stable ligand-receptor interactions and productive binding modes. ^39^ Thus, the observed values suggest that Peptide A maintains a stable conformation when bound to these cytokines, with potential for biologically relevant modulation. These molecular dynamics findings support the hypothesis that Peptide A exerts dual immunomodulatory functions by stabilizing interactions with both anti-inflammatory and pro-inflammatory cytokines. Such bifunctional receptor engagement strengthens its candidacy as a therapeutic agent in conditions like placental syndromes.

## Conclusion

In summary, our in silico study on peptides derived from the coelomic fluid of *Echinometra lucunter* highlights their potential to target key inflammatory cytokines involved in placental syndromes. Peptide A was selected due to its favorable ADMET properties and strong binding to pro-inflammatory cytokines, such as TRAIL, MIF, IL-1β, and PDGF. It also showed promising interactions with anti-inflammatory targets, including IL-10 and c-Met. Molecular dynamics simulations of the peptide-receptor complexes revealed that Peptide A formed stable interactions with IL-10 and IL-1β, confirming the stability of the complexes. These findings suggest that IL-10 and IL-1β are the most relevant targets for Peptide A. Given the promising binding profiles and stable interactions, Peptide A could potentially serve as a dual-function therapeutic in placental syndromes. Nonetheless, experimental validation is necessary to confirm its efficacy and safety in treating placental dysfunctions.

## Data Availability

Zenodo: Exploring the Potential of Peptides Derived from the Coelomic Fluid of Echinometra lucunter Targeting Inflammatory Cytokines in Placental Syndromes: In Silico Study https://doi.org/10.5281/zenodo.17230998. ^40^ This project contains the following underlying data:
• HGF LUCUNTER.xlsx • IL10 LUCUNTER.xlsx • ILB LUCUNTER.xlsx • MIF LUCUNTER.xlsx • PDGF LUCUNTER.xlsx • TRAIL LUCUNTER.xlsx Data is available under the terms of the CC BY 4.0 ^40^
