# Supplementary material for: Exploring the Potential of Peptides Derived from the Coelomic Fluid of Echinometra lucunter Targeting Inflammatory Cytokines in Placental Syndromes: In Silico Study
Source: F1000Res. 2025 Nov 6;14:1060. Originally published 2025 Oct 10. [Version 2] doi: 10.12688/f1000research.166678.2 (PMC13580618; doi:10.12688/f1000research.166678.2)
Supplement: Peer review history

# Reviewer report: Reviewer response for version 2

**DOI:** 10.5256/f1000research.190867.r431347

**Published:** 2025-12-10

**Authors:** Norbertus Marcell Prayogi

**Affiliations:** 1 Medical Faculty, Universitas Lampung, Bandar Lampung, Lampung, Indonesia

​The manuscript presents a scientifically sound and well-executed study that effectively integrates medical insights with bioinformatics analysis. The authors have succeeded in drafting a paper that is not only logically organized but also methodologically robust. A standout feature of this work is the transparency of the bioinformatics pipeline; the computational methods, algorithm parameters, and data processing steps are described with sufficient detail to ensure reproducibility. This clarity allows other researchers to easily replicate the in-silico experiments and verify the validity of the results using the provided datasets.

 ​Furthermore, the results are presented clearly, with the biological interpretation aligning well with the statistical outputs. The findings offer valuable implications for the medical field and demonstrate a high potential for consistent results upon replication. Given the quality of the writing and the rigor of the study design, I find no significant flaws in the manuscript. I recommend this article for indexing in its current form, as it makes a solid and reliable contribution to the literature.

 ​Recommendation: Accept.

Is the work clearly and accurately presented and does it cite the current literature?

Yes

If applicable, is the statistical analysis and its interpretation appropriate?

Not applicable

Are all the source data underlying the results available to ensure full reproducibility?

Yes

Is the study design appropriate and is the work technically sound?

Yes

Are the conclusions drawn adequately supported by the results?

Yes

Are sufficient details of methods and analysis provided to allow replication by others?

Yes

Reviewer Expertise:

Bionformatics study and internal medicine and physiology

I confirm that I have read this submission and believe that I have an appropriate level of expertise to confirm that it is of an acceptable scientific standard.

# Reviewer report: Reviewer response for version 2

**DOI:** 10.5256/f1000research.190867.r430430

**Published:** 2025-11-11

**Authors:** Aldi Tamara Rahman

**Affiliations:** 1 Dopamine Science Institute, Depok, Indonesia

I find the revised version acceptable for publication

Is the work clearly and accurately presented and does it cite the current literature?

Yes

If applicable, is the statistical analysis and its interpretation appropriate?

Not applicable

Are all the source data underlying the results available to ensure full reproducibility?

Yes

Is the study design appropriate and is the work technically sound?

Yes

Are the conclusions drawn adequately supported by the results?

Yes

Are sufficient details of methods and analysis provided to allow replication by others?

Yes

Reviewer Expertise:

Cell biology, Bioinformatics

I confirm that I have read this submission and believe that I have an appropriate level of expertise to confirm that it is of an acceptable scientific standard.

# Reviewer report: Reviewer response for version 1

**DOI:** 10.5256/f1000research.183702.r423438

**Published:** 2025-10-28

**Authors:** Aldi Tamara Rahman

**Affiliations:** 1 Dopamine Science Institute, Depok, Indonesia

This study presents an interesting approach to identifying new drug candidates derived from marine organisms. Although the research is still conducted through an in silico approach, the analyses involved are relatively complex, including molecular dynamics simulation. However, there are several points that the authors should consider to improve the quality of this manuscript:

 Please ensure that the manuscript follows proper and consistent scientific English terminology, for example, the use of “preeclamptic” versus “pre-eclamptic,” and similar terms.

 The molecular docking method used should be validated through redocking between the native ligand and its receptor. Please ensure that, after redocking, the resulting RMSD value is < 2 Å to confirm docking reliability.

 The membrane permeability analysis should include graphical representations of the energy profile and the compound’s distance relative to the membrane, rather than relying solely on visual illustrations of the compound penetrating the cell membrane.

Is the work clearly and accurately presented and does it cite the current literature?

Yes

If applicable, is the statistical analysis and its interpretation appropriate?

Not applicable

Are all the source data underlying the results available to ensure full reproducibility?

Yes

Is the study design appropriate and is the work technically sound?

Yes

Are the conclusions drawn adequately supported by the results?

Yes

Are sufficient details of methods and analysis provided to allow replication by others?

Yes

Reviewer Expertise:

Cell biology, Bioinformatics

I confirm that I have read this submission and believe that I have an appropriate level of expertise to confirm that it is of an acceptable scientific standard, however I have significant reservations, as outlined above.
